# Severe pancytopenia and splenomegaly associated with felty's syndrome, both fully responsive solely to corticosteroids

**DOI:** 10.1002/ccr3.1396

**Published:** 2018-01-31

**Authors:** George D. Liatsos, Ioanna Tsironi, Dimitrios Vassilopoulos, Spyridon Dourakis

**Affiliations:** ^1^ 2nd Academic Department of Internal Medicine National and Kapodistrian University of Athens School of Medicine Hippokration General Hospital Athens Greece; ^2^ Joint Rheumatology Program, Clinical Immunology‐Rheumatology Unit 2nd Department of Medicine and Laboratory National and Kapodistrian University of Athens School of Medicine Hippokration General Hospital Athens Greece

**Keywords:** corticosteroids, Felty's syndrome, pancytopenia, rheumatoid arthritis, Sjorgen's syndrome, splenomegaly

## Abstract

In severe cases of pancytopenia with subsequent infections due to long‐term untreated Felty's syndrome, the initiation of immunosuppressive treatment with sole prednisone (1 mg/kg *iv*) should be considered, despite that, the low neutrocytes count would make one physician hesitant. A full resolution of whole blood count within 3 weeks and a 30% reduction in spleens sized was noted.

A 73‐year‐old Caucasian female with long‐standing RA was admitted with fever, abdominal pain, and bloody mucous diarrheas. On clinical examination, her abdomen was soft with tenderness and resistance of the left iliac fossa on palpation and palpable spleen. Metacarpophalangeal and metatarsophalangeal joints had signs of synovitis. Examination of the other systems was unremarkable. Laboratory findings showed severe neutropenia (Table 1). Her medical history was important for discontinuation of hydroxychloroquine because of eye disorders, and of methotrexate due to anemia, three years ago, when also retired from her follow‐up.

**Table 1:** Patient's laboratory test results during hospitalization and follow‐up (abnormal values in bold)

Blood test	Admission	Day 2 *Filgastrim initiation*	Day 7	Day 12 *Prezolon initiation*	Day 20 (discharge)	2 weeks after discharge
WBC count/μL	**1090**	**960**	**980**	**1350**	**1180**	6400
Neutrocytes %	**25**	**10**	**20**	**28**	**18**	52
Lymphocytes %	**47**	35	**44**	33	**68**	29
Monocytes %	**25**	**55**	**30**	**22**	**14**	**17**
Atypical forms%	**3**	0	**6**	**13**	0	**2**
Hct %	**29.6**	**28.2**	**28.8**	**25.9**	**27,4**	35.8
Hb g/dL	10.2	10.1	9.7	8.2	9.3	12.0
PLT count/μL	150,000	140,000	**70,000**	**34,000**	200,000	297,000
Urea mg/dL	**51**	26	12	25	**67**	**95**
Creatinine mg/dL	**1.2**	**1.5**	0.8	0.8	0.8	1.0
LDH IU/L	207	161	**239**	**300**	185	**265**
AST IU/L	16	42	18	17	12	12
ALT IU/L	12	15	8	14	8	14
*γ*‐GT IU/L	15	15	24	16	23	31
ALP IU/L	48	43	47	49	40	55
TBIL mg/dL	0.8	0.8	0.8	0.9	0.9	1.0
ESR mm/1 h	**65**					40
CRP mg/L	**152.5**	**103.3**	**36**	10.2	2.7	12.7
Ferritin μg/L	**649.5**	–	–	–	–	–
Anti‐HIV^a^,^b^	neg	–	–	–	–	–
HBsAg	neg	–	–	–	–	–
Anti‐HAV IgM	neg					
Anti‐HCV^a^	neg					
Blood and urine cultures	neg	neg	neg	neg		
Serum screening for auto‐antibodies						
ANA, anti‐dsDNA	neg					
c‐, p‐ANCA	neg					
AMA, anti‐ENA (Elisa)	neg					
A.S.M.A.	pos					
Anti‐CCP3 U/mL (Elisa)	>1600					
IgG mg/dL	1244	–	–	–	–	–
IgA mg/dL	133	–	–	–	–	–
IgM mg/dL	105	–	–	–	–	–
C3c mg/dL	81	–	–	–	–	–
C4 mg/dL	15	–	–	–	–	–
Ra test IU/mL	**51.3 (<15)**	–	–	–	–	–
Immunofixation assay	No monoclonal globulin					
Stool analysis						
Leukocytes	**Many**					
Erythrocytes	**Many**					
Parasites	neg					
Cultures	Normal flora					
*Chlostridium difficile* antigen	neg					
*Chlostridium difficile* toxin	neg					
Bone marrow biopsy^b^						
Blood flow cytometry^c^						
Spleen's size	**17 cm**					12 cm

a Laboratory evaluation for other pathogens: Antibodies against *Brucella sp*. neg, antibodies against K39 *Leishmania infantum* antigen: neg.

b Bone marrow histology was consistent with a wide range of secondary lesions including peripheral blood cell and leukocyte destruction, infections, or other systemic diseases, thus excluding any hematological disease.

c blood flow cytometry showed normal lymphocyte subpopulations and increased the percentage of activated T‐lymphocytes thus excluding large granular lymphocytic (LGL) leukemia.

The patient was introduced to antimicrobial treatment (ciprofloxacin + metronidazole) and was subjected to bone marrow (bm) aspiration. Myelogram showed a hypercellular bone marrow, without blasts or infiltration, while histology was consistent with a wide range of secondary lesions including peripheral blood cell and leukocyte destruction, infections, or other systemic diseases, thus excluding any hematological disease. Furthermore, blood and bm flow cytometry showed normal lymphocyte subpopulations and increased the percentage of activated T‐lymphocytes. Thus, she was started on filgastrim. On day four, diarrheas subsided and renal function restored. Chest and abdomen computed tomography showed hepatomegaly (20 cm), splenomegaly (17 cm), and extensive concentric bowel wall thickening of the sigmoid colon and rectum with coexistent diverticulae and pericolic fat stranding, findings consistent with colitis and diverticulitis.

Two weeks after admission, marked neutropenia was still present, despite the daily administration of G‐CSF. PLT count on the other hand gradually dropped to 30,000/μL. We made the decision for the initiation of corticosteroid immunosuppressive therapy before she developed any possible life‐threatening hemorrhage, despite severe neutropenia. Thus, filgastrim was discontinued, and the patient was started on prednisolone (1 mg/kg of body weight *iv*). Furthermore, due to xerophthalmia and xerostomia with subsequent pathologic tear film break up time, the patient was subjected to a lower lip biopsy which showed focal lymphocytic sialadenitis of 4th‐grade. When PLT count started rising, she was subjected to colonoscopy that revealed multiple diverticula orifices that deformed the bowel lumen of the sigmoid colon. She was finally discharged after three weeks of hospitalization (Table 1) under methylprednisolone 32 mg daily and a very close outpatient follow‐up.

On a new blood examination two weeks after discharge, pancytopenia, surprisingly, had completely resolved (Table 1). She was started on methotrexate and methylprednisolone was tapered. Two months later, complete blood count remained normal, while spleen's size had been decreased to 12 cm. Her medication was modified to increased methotrexate dose and gradual methylprednisolone taper.

Most authors regard FS as a severe variant developing in <1% of RA patients 1, 2. It usually appears in the course of long‐standing RA and is characterized by neutropenia and splenomegaly. RF, anti‐CCP positivity, and rheumatoid nodules occur much more often in FS. Other associations of FS are with liver involvement, increased risk for malignancies and several extra‐articular features 2.

As a result of neutropenia, the most common and most important feature of FS, patients are increasingly susceptible to infections, with higher incidence of bacterial infections when neutrophil count is lower than 500/μL, *Herpes zoster* and fungi 2. Most common infections affect the skin, mouth, upper and lower respiratory, and urinary tracts 1, 2. However, our patient presented with clinical, radiological, and endoscopic findings consistent with colitis and diverticulitis. Probable infection was successfully treated with a week antibiotical administration. To our knowledge, this is the first reported presentation of FS with colitis/diverticulitis.

Anemia in FS is the same with the chronic anemia of RA patients, as in our patient. However, she developed severe thrombocytopenia. In FS, thrombocytopenia is considered to be present 2, but not at such low count as measured in our case (30,000PLT/μL). In addition, the ten‐day administration of G‐CSF is a factor that among other side effects, is considered to cause rarely thrombocytopenia 3. Thrombocytopenia resolved promptly with corticosteroids administration, thus favoring immunologic etiology. Concerning immunological features, RF is positive in 95–100% of the patients 2, in high titers while in our patient it was threefold increased. ANA positivity is also common (47–100%) but was negative in our patient. On the other hand, anti‐CCP3 titers were too highly measured, as expected. Sjogren's syndrome was established by histology.

In 30% of FS patients, there is evidence of LGL expansion. Similar LGL expansion has been noticed in a percentage of RA without FS 1, 2. In our case, blood and bm flow cytometry, and bm histology, excluded LGL, as well as other possible hematological diseases. In addition, lower lip biopsy excluded possible MALT lymphoma.

Methotrexate is the most widely administered drug in patients with RA and neutropenia 4, while Cyclosporine is a second‐line drug and Cyclophosphamide is an alternative. Corticosteroid therapy is not considered as the treatment of neutropenia in FS or RA 4, and has been used in case reports and only in combination with other regimens 1. The pathogenesis of neutropenia in FS is unclear and complicated. Autoimmune process is being studied. Auto‐antibodies against the eukaryotic elongation factor‐1A‐1, that is, expressed in myeloid cell precursors are present in 66% of FS patients, suggesting a possible role of auto‐antibodies indirectly inhibiting granulopoiesis or enhancing the clearance of neutrophils 5. Thus, it is possible that an autoimmune process which leads to the production of RF auto‐antibodies, or other auto‐antibodies that activate neutrophils, overtime contributes to FS neutropenia as a consequence of immune complex‐driven neutrophil depletion 5. Corticosteroids in our case resulted in restoring WBC count, and interestingly, in a reduction in spleen's size by 5 cm after two months a finding never reported elsewhere in the literature. The spleen may be an important organ that, following neutrophil activation in the periphery, performs multiple FS‐specific roles, including neutrophil sequestration and auto‐antibody induction and selection 5.

We report a unique infectious presentation of untreated RA who developed FS and Sjorgen's syndrome with colitis/diverticulitis. The main purpose of the article, however, is to underline the fundamental new information of prompt initiation of sole corticosteroid treatment for pancytopenia, a potentially life‐threatening complication, despite the fact that severe neutropenia would make any physician hesitant to receive such a decision.

## Conflict of Interest

No one of the authors of the present article have any conflict of interests to declare.

## Authorship

All authors had access to the data and a role in writing the manuscript.
